# Supplementary material for: Multi-Modal in Vitro Experiments Mimicking the Flow Through a Mitral Heart Valve Phantom
Source: Cardiovasc Eng Technol. 2024 May 23;15(5):572–83. doi: 10.1007/s13239-024-00732-3 (PMC11582118; doi:10.1007/s13239-024-00732-3)
Supplement: Supplementary file 1 — Supplementary Material 1 [file 13239_2024_732_MOESM14_ESM.pdf]

MULTI-MODAL IN VITRO EXPERIMENTS MIMICKING THE FLOW THROUGH A MITRAL HEART VALVE PHANTOM

Lea Christierson[1,2], Petter Frieberg[3], Tania Lala[2,3], Johannes Töger[3], Petru Liuba[1], Johan Revstedt[4], Hanna Isaksson[2], Nina Hakacova[1]

[1]Department of Clinical Sciences Lund, Pediatric Heart Center, Skåne University Hospital, Lund University, Lund, Sweden,
[2]Department of Biomedical Engineering, Lund University, Lund, Sweden,
[3]Department of Clinical Sciences Lund, Clinical Physiology, Skåne University Hospital, Lund University, Lund, Sweden,
[4]Department of Energy Science, Lund University, Lund, Sweden

CORRESPONDING AUTHOR: Lea Christierson, lea.christierson@med.lu.se

SUPPLEMENTARY MATERIAL

The tensile tests, setup

To quantify the material properties of the silicone material for future material modeling purposes, material tests were performed on N = 6 dog bone samples of stiffness Shore A25 and A40, and N = 5 samples of Shore A60. Uniaxial tensile tests with a preload of 1 N and a loading rate of 6 mm/s, corresponding to a 10 %/s strain rate, were performed until a total of 50 mm displacement was reached. The thickness of the dog bones was the same as the thickness of the mitral valves and the loading rate corresponded to the average loading rate of the valve in the phantom. The dimensions of the dog bone are reported in Fig S.1a.

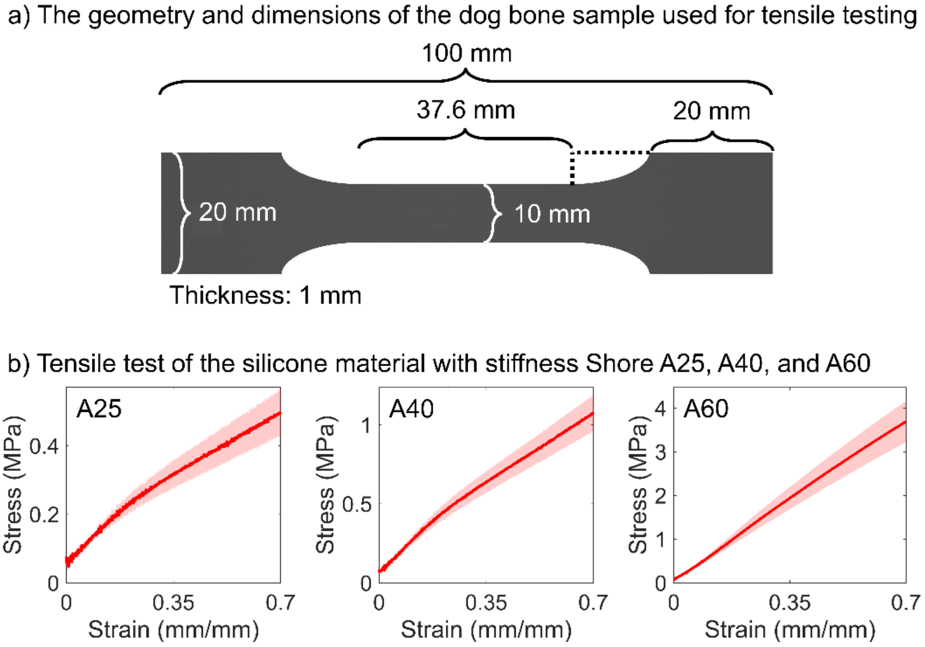

Fig. S.1 *a) The dimensions of the dog bone samples used for material testing. The shape of the dog bone is symmetric about its two in-plane center axes. b) Uniaxial tensile test performed on dog bones of stiffness Shore A25, A40, and A60, respectively. The data is averaged over the number of samples with the mean value shown as a red line and the standard deviation plotted as a pink shadow.*

Results

The tensile tests of the dog bone samples show a bilinear behavior of the silicone material for all three stiffnesses, with the transition region occurring around strains of 0.1-0.3. For strains lower than 0.1, Young's moduli were 0.91 MPa, 1.89 MPa, and

5.13 MPa for the three different materials. Further, for strains greater than 0.3 Young's moduli corresponded to 0.51 MPa, 1.25 MPa, and 5.26 MPa (Fig. S.1b).

MRI parameters used for imaging

The phantom was analyzed using cine MRI and 2D and 4D PC-MRI. An overview of all MRI sequence parameters is provided in Table S.1.

Table S.1 *An overview of all MRI sequence parameters.*

	**Cine MRI**	**2D PC-MRI**	**4D PC-MRI**
Scan duration (s)	45	168	432
Field of view, phase x read x slice (mm^3)	343 x 377 x 4	324 x 400 x 5	216 x 240 x 108
Spatial resolution, acquired, base x phase, slice (mm^3)	0.87 x 0.87 x 4	1.9 x 1.9 x 5	3 x 3 x 3
Spatial resolution, reconstructed, base x phase, slice (mm^3)	0.87 x 0.87 x 4	1.9 x 1.9 x 5	3 x 3 x 3
Temporal resolution, acquired (ms)	17.9	9.8	44
Temporal resolution, reconstructed (ms)	17.9	28.7	40.4
Slice thickness (mm)	4	5	3
TR / TE / flip angle (ms / ms / °)	3.5 / 1.47 / 40	4.9 / 2.47 / 20	5.5 / 2.7 / 32
Venc (cm/s)	n/a	200	150
Acceleration	none	none	iPat = 3

Measuring the opening of the valve

The valve opening was quantified for all six flow cases based on measurements on cine MRI images (Fig. S.2) and 2D and 3D Echo images (Fig. S.3).

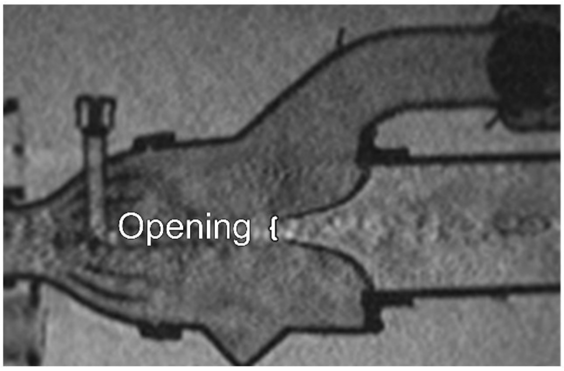

Fig. S.2 *The valve opening, defined as a distance, measured on cine MRI images. The cine MRI image demonstrates where and how the distance measurements were conducted.*

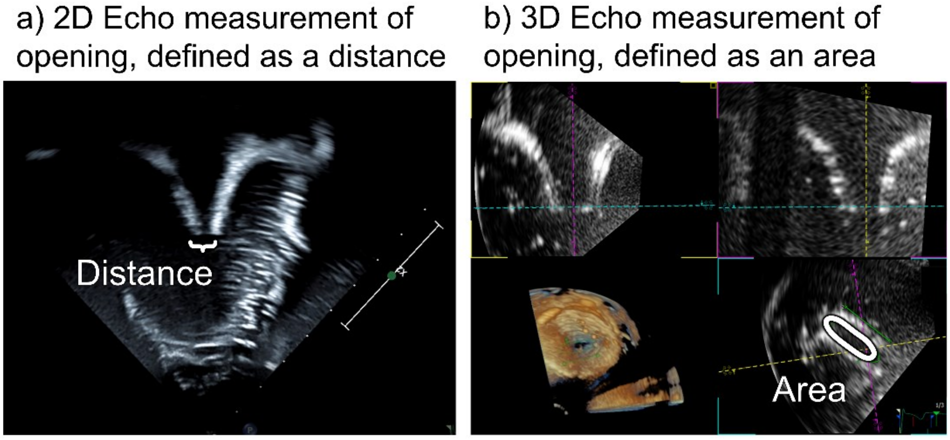

Fig. S.3 *Demonstration of where and how the manual measurements of the valve opening (a) defined as a distance and (b) defined as an area measured on 2D and 3D Echo data were performed.*

Complementary graphs of measurements

In the following figures, the complete set of measurements for all parameters and investigated cases can be seen, such as the atrial and ventricular pressure (Fig. S.4), the velocity measured by 2D and 4D PC-MRI (Fig. S.5) and the velocity profiles (Fig. S.6).

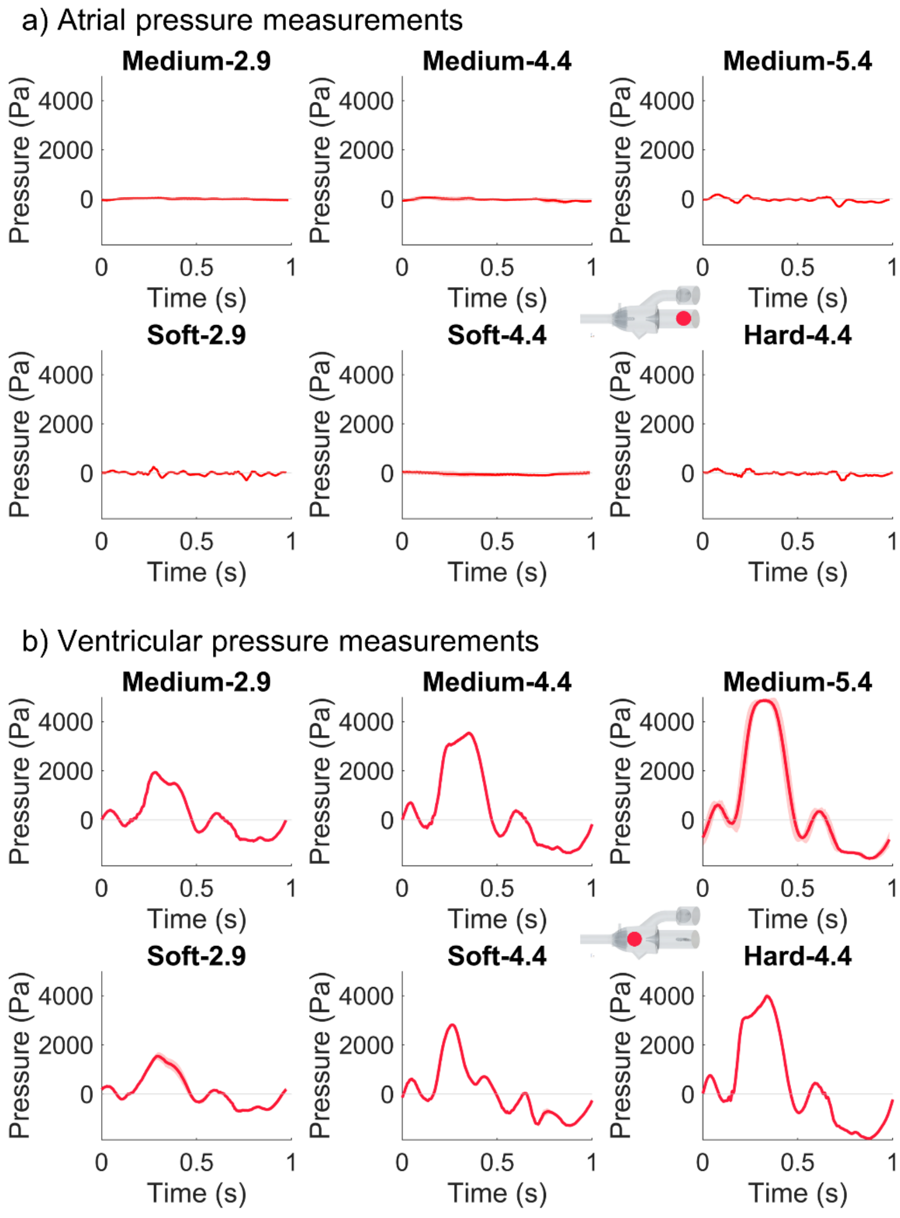

Fig. S.4 *The a) atrial and b) ventricular pressure measurements for all investigated cases. The red line represents the average pressure, and the pink background the standard deviation*

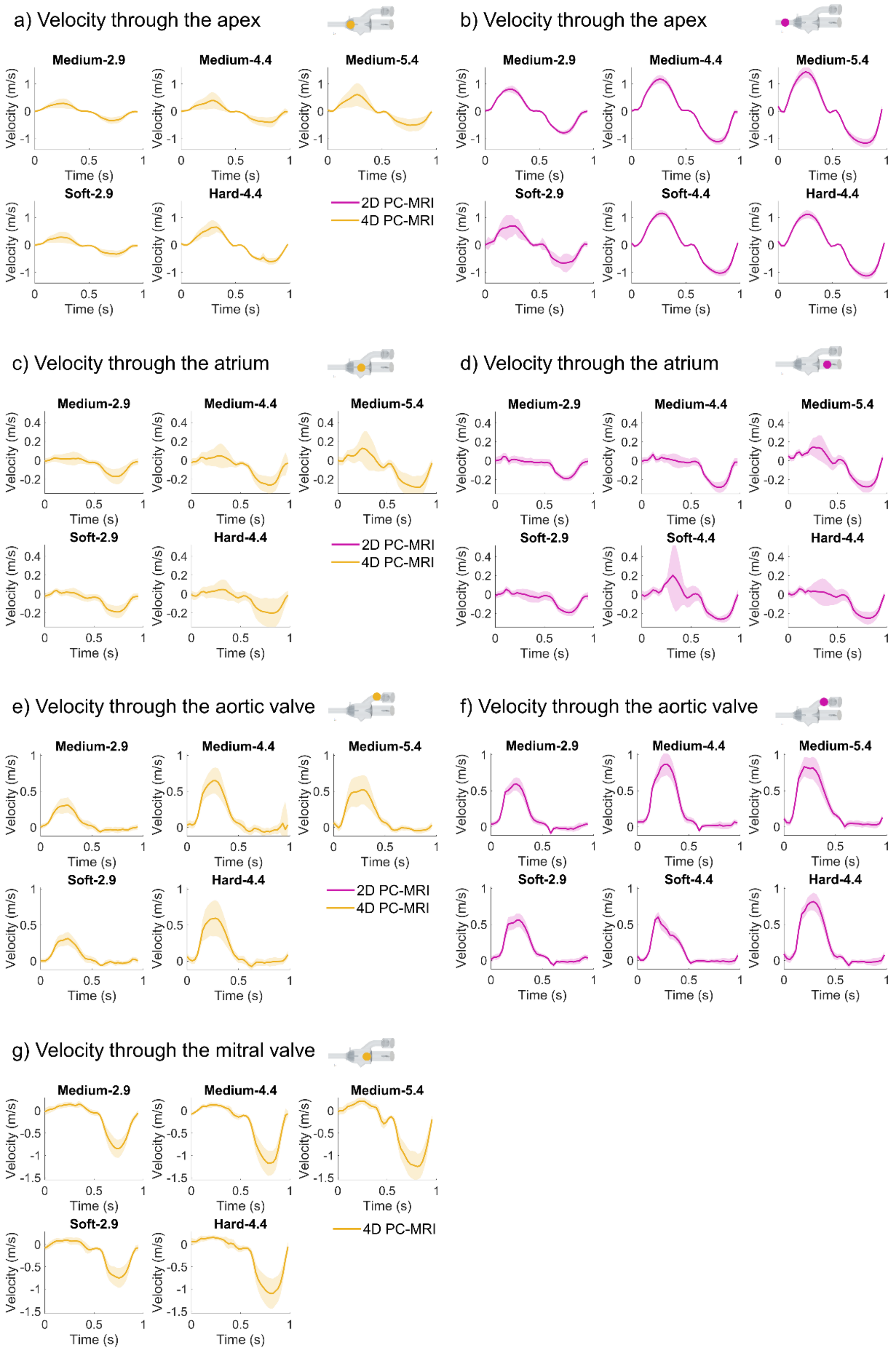

Fig. S.5 *The 2D and 4D PC-MRI velocity measurements at the apex, atrium, aortic valve, and mitral valve for all six cases. The measurements show the spatially averaged velocity (dark pink/yellow) across the region of interest with the standard deviation (light pink/yellow shade).*

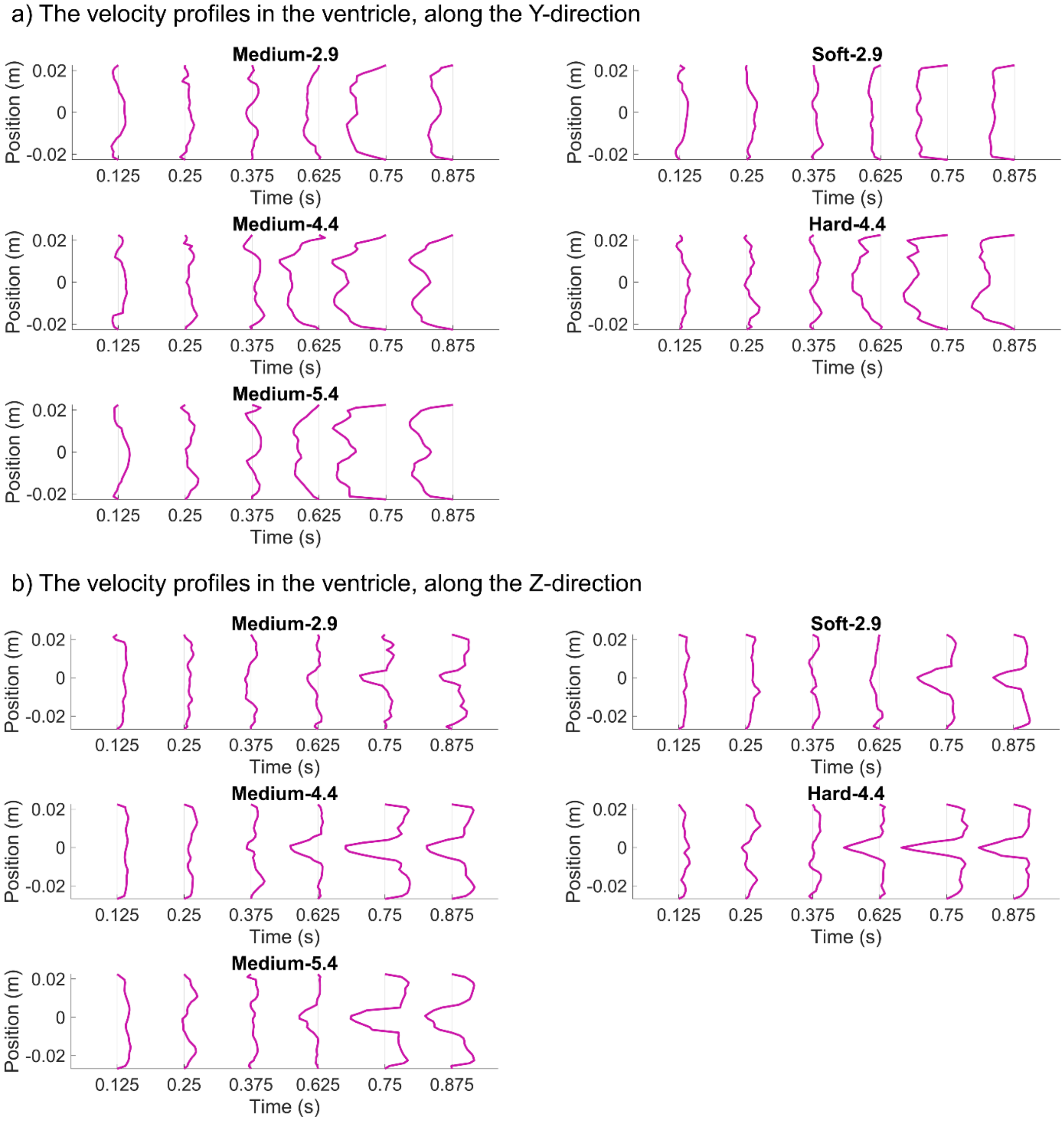

Fig. S.6 *The velocity profile in the ventricle along lines in the a) y-direction and b) z-direction.*
